# Rho-Rho kinase pathway in the actomyosin contraction and cell-matrix adhesion in immortalized human trabecular meshwork cells

**Published:** 2011-07-14

**Authors:** C. Ramachandran, R.V. Patil, K. Combrink, N.A. Sharif, S.P. Srinivas

**Affiliations:** 1 School of Optometry, Indiana University, Bloomington, IN; 2 Pharmaceutical Research, Alcon Research Ltd., Fort Worth, TX

## Abstract

**Purpose:**

The outflow facility for aqueous humor across the trabecular meshwork (TM) is enhanced by agents that oppose the actomyosin contraction of its resident cells. Phosphorylation of MYPT1 (myosin light chain [MLC] phosphatase complex of Type 1) at Thr853 and Thr696 inhibits dephosphorylation of MLC, leading to an increase in actomyosin contraction. In this study, we examined the effects of Rho kinase (ROCK) inhibitors on the relative dephosphorylation of the two sites of MYPT1 using human TM cells (GTM3).

**Methods:**

Dephosphorylation of MYPT1 at Thr853 and Thr696 was determined by western blot analysis following exposure to selective inhibitors of ROCK, namely Y-27632 and Y-39983. Consequent dephosphorylation of MLC and decreases in actomyosin contraction were assessed by western blot analysis and collagen gel contraction assay, respectively. Changes in the cell-matrix adhesion were measured in real time by electric cell-substrate impedance sensing and also assessed by staining for paxillin, vinculin, and focal adhesion kinase (FAK).

**Results:**

Both ROCK inhibitors produced a concentration-dependent dephosphorylation at Thr853 and Thr696 of MYPT1 in adherent GTM3 cells. IC_50_ values for Y-39983 were 15 nM and 177 nM for dephosphorylation at Thr853 and Thr696, respectively. Corresponding values for Y-27632 were 658 nM and 2270 nM. Analysis of the same samples showed a decrease in MLC phosphorylation with IC_50_ values of 14 nM and 1065 nM for Y-39983 and Y-27632, respectively. Consistent with these changes, both inhibitors opposed contraction of collagen gels induced by TM cells. Exposure of cells to the inhibitors led to a decrease in the electrical cell-substrate resistance, with the effect of Y-39983 being more pronounced than Y-27632. Treatment with these ROCK inhibitors also showed a loss of stress fibers and a concomitant decrease in tyrosine phosphorylation of paxillin and FAK.

**Conclusions:**

Y-39983 and Y-27632 oppose ROCK-dependent phosphorylation of MYPT1 predominantly at Thr853 with a corresponding decrease in MLC phosphorylation. A relatively low effect of both ROCK inhibitors at Thr696 suggests a role for other Ser/Thr kinases at this site. Y-39983 was several-fold more potent when compared with Y-27632 at inhibiting the phosphorylation of MYPT1 at either Thr853 or Thr696 commensurate with its greater potency at inhibiting the activity of human ROCK-I and ROCK-II enzymes.

## Introduction

The outflow of aqueous humor across the trabecular meshwork (TM) is regulated by, among other factors, actomyosin contraction of the resident TM cells and altered extracellular matrix (ECM) [1-3]. Ex vivo perfusion studies have demonstrated that agents that increase the actomyosin contraction of TM cells decrease aqueous humor outflow and vice versa [4-6]. These observations led to the hypothesis that the contraction of TM cells regulates the outflow facility, possibly through the reorganization of the TM through altered cell-ECM interactions.

Actomyosin contraction is dependent on the phosphorylation of the regulatory light chain of myosin II (also called the myosin light chain or MLC; 20 kDa). MLC is phosphorylated at its Ser19 and/or Thr18 residues by MLC kinase (MLCK), which is a (Ca^2+^-calmodulin)-dependent kinase [7]. Accordingly, G protein-coupled receptors (GPCRs) that mobilize intracellular-free Ca^2+^ ([Ca^2+^]_i_) activate MLCK and induce MLC phosphorylation. However, sustained contraction is dependent on the activity of MLC phosphatase (MLCP) [8-10]. Investigations in the last decade, notably of smooth muscle cells, have unraveled the molecular aspects related to the regulation of MLCP [11,12]. It is now known that MLCP is a complex of three subunits: a regulatory/myosin binding subunit (MYPT1), a catalytic subunit (PP1cδ), and M20 [12]. The MLCP activity is regulated through MYPT1 phosphorylation by many kinases, including integrin-linked kinase (ILK), protein kinase C (PKC), ZIP kinase, and Rho-associated coiled-coil-containing protein kinase (ROCK) [13]. In a variety of cell types, ROCK is known to inhibit the phosphatase activity of MLCP by phosphorylating MYPT1 at Thr696 and Thr853 [14,15]. However, differences in the correlation between the site of MYPT1 phosphorylation and the extent of MLC phosphorylation and/or force generation have also been documented [16,17]. Given the important role of ROCK in the regulation of actomyosin contraction, there is significant interest in employing its inhibitors to facilitate outflow across the TM [18,19], and thus ROCK inhibitors are of special interest as potential ocular hypotensive agents.

In this study, we investigated the molecular targets of ROCK on actomyosin contraction in TM cells. Specifically, we focused on establishing the relative significance of phosphorylation of MYPT1 by ROCK at Thr696 as compared with Thr853. Our approach involved challenging a human TM cell line with two relatively selective inhibitors of ROCK, followed by assaying the degree of dephosphorylation of the two inhibitory sites. These inhibitors, namely, Y-27632 and Y-39983, are known to increase the outflow facility across TM [19-21]. We confirmed the impact of the dephosphorylation downstream in terms of MLC phosphorylation, actomyosin contraction, and cell-matrix adhesion. Our results show that the predominant inhibitory phosphorylation site of MYPT1 regulated by ROCK is Thr853. Inhibition of phosphorylation at this site correlates with a decrease in MLC phosphorylation as well as in actomyosin contraction. As a consequence of the latter, the inhibition of ROCK also results in a loss of cell-ECM adhesion, which may increase the aqueous humor outflow facility deemed useful for lowering intraocular pressure (IOP).

## Methods

### Drugs and chemicals

MYPT1, phospho-MYPT1 (Thr853), phospho-MLC (Thr18 and Ser19), phospho-paxillin (Tyr118), and FAK (Tyr397) antibodies were obtained from Cell Signaling Technology (Danvers, MA). Phospho-MYPT1 (Thr696) antibody was bought from Millipore (Temecula, CA). Anti-paxillin antibody was purchased from BD biosciences (San Jose, CA), and anti-vinculin antibody, along with blebbistatin and Y-27632, from Sigma (St. Louis, MO). Y-39983 was synthesized at Alcon Research, Ltd. (Fort Worth, TX). Texas-Red conjugated phalloidin, Alexa-488 conjugated goat-anti mouse, and anti-rabbit antibodies were purchased from Molecular Probes (Eugene, OR). Gold electrodes (8W10E+) for measuring electrical cell-substrate resistance (ECSR) were purchased from Applied Biophysics, Inc. (Troy, NY), and the SuperScript III Cells Direct cDNA Synthesis Kit was from Invitrogen (Grand Island, NY).

### Cell culture

Cell cultures of an immortalized human glaucomatous trabecular meshwork (GTM3) cell line were grown at 37 °C in 5% CO_2_ in Dulbecco’s Minimum Essential Medium (DMEM + Glutamax) supplemented with 10% fetal bovine serum and 10 μg/ml of gentamicin (Invitrogen, Grand Island, NY) as previously described [22]. Upon reaching confluence, cells were divided using 0.05% trypsin. Cells of passages 13 to 20 were used in our experiments.

### Western blot analysis of MYPT1 and MLC phosphorylation

Following treatment with the desired drugs/agents, cells were rinsed in PBS and solubilized in 300 µl of 2× Laemmli sample buffer. The lysate was sonicated briefly and boiled for 5 min. Equal amounts of protein (30 μg) were loaded in 8% or 12% SDS–PAGE gels. Following electrophoresis, proteins were transferred to a nitrocellulose membrane (Biorad, Hercules, CA), blocked with 5% fat-free milk for 1 h, and incubated with specific phospho-MYPT1 or phospho-MLC antibodies overnight. The membranes were next incubated with the appropriate secondary antibodies for 1 h. The blots were finally washed and developed using an enhanced chemiluminescence kit (Pierce, Rockford, IL) according to the manufacturer’s instructions.

### Immunofluorescence

Cells were first treated for the indicated time period in the serum-rich medium described earlier. Following a brief rinse with PBS, cells were fixed with 4% paraformaldehyde for 10 min and then permeabilized with 0.1% Triton-X for 10 min. For phosphorylated MLC staining, cells were blocked with 10% goat serum + 3% BSA, and, for vinculin and paxillin staining, the cells were blocked with 10% FBS for 1 h. Cells were incubated in primary antibody overnight, followed by secondary antibody incubation for 1 h. Double staining for actin was performed by incubating cells in Texas-red conjugated phalloidin for 20 min. After extensive washing, coverslips were mounted using ProLong AntiFade (Molecular Probes, Eugene, OR). Images were acquired using a Leica SP5 (Leica Microsystems, Bannockburn, IL) confocal microscope. After similar treatments, DIC images of cells fixed on coverslips were also obtained using a Nikon E800 (Nikon Instruments, Melville, NY) microscope.

### Collagen gel contraction assay

A collagen gel contraction assay was performed as previously described [23,24]. The wells of 24-well culture plates were coated with 1% BSA at 37 °C for 1 h. Glaucomatous trabecular meshwork cells were trypsinized and resuspended in culture medium at a density of 1×10^7^ cells/ml. Rat-tail collagen Type I (BD Biosciences, San Jose, CA), 10× DMEM (Sigma, St. Louis, MO), reconstitution buffer (pH 7.3), TM cell suspension, and distilled water were mixed on ice to obtain a final concentration of 1.9 mg/ml of collagen and a final cell density of 2×10^5^ cells/ml. The resultant mixture (0.5 ml) was added to each well of the BSA-coated culture clusters, and collagen gel formation was induced by incubation at 37 °C for 90 min. Serum-rich or serum-free DMEM (0.5 ml), without or with the drugs, was then added on top of the gels. After 1 h, the gels were freed from the walls of the culture wells. They were subsequently imaged every 24 h for 2 days. The area was calculated using NIH ImageJ software.

### Reverse transcription polymerase chain reaction

Total RNA was isolated using Trizol reagent (GIBCO BRL, Grand Island, NY) and quantified by measuring absorption at 260 nm. Genomic DNA contamination was removed by treating the extraction with DNase I. First-strand cDNA synthesis and PCR amplification of cDNA was performed using the Superscript III CellsDirect cDNA Synthesis Kit (Invitrogen, Carlsbad, CA). Reverse transcription polymerase chain reaction (RT–PCR) products were run on a 2% agarose gel and visualized by ethidium bromide staining along with 100 bp markers (Amersham Biosciences, Piscataway, NJ). The primer sequences and expected product sizes are given in Table 1. The products were confirmed with corresponding negative controls performed for all the primer pairs employed.

**Table 1:** Primer sequences.

**Gene ID**	**Primer sequence**	**Ta**	**Size (bp)**	**References**
Myosin IIA	Sense: GAAGGTCATCCAGTATCTGGCG	52	355	[46]
	Antisense: ACAGGAAGCGGTATTTGTTGTACG			
Myosin IIB	Sense: AGAAGGGCATGTTTCGTACCG	52	236	[46]
	Antisense:TGAATTCCTGGAAAACTATTCGGTTAG			
Myosin IIC	Sense: AAGCCATTGTGGAGATGTACCG	52	386	[46]
	Antisense: GGTAGGTCTCAATGTTGGCGC			
MYPT1	Sense: CCGTATTGAATCTCTGGAACAAG	55	308	[42]
	Antisense: TTGCAGGAGACTCATCTTTTCTC			
MYPT2	Sense: GAAGGTGAAGATGAAGCTTCTGA	60	240	[42]
	Antisense: AGTTTTTCTCAGTCCCAATCTCC			
ROCK1	Sense: CTCCGAGACACTGTAGCACCAGTT	63	328	[42]
	Antisense: TTTGAGGTTCTGCACTTCTGCTCC			
ROCK2	Sense: TTAAGGAAAACCCAGGCAGAAGT	60	375	[42]
	Antisense: TTCTTCTTGTTCTAGGCTCTGCTG			
EC-MLCK	Sense: AGGTGCTTCAGAATGAGGACGTGT	56	130	[43]
	Antisense: TGTAGCATCAGTGACACCTGGCAA			
SM-MLCK	Sense: TGAGCTGTTCGAGCGCATCATT	52	167	[10]
	Antisense:TGGTGCCTGTCTTGTTGACACA			

### Electrical cell-substrate impedance sensing

Cells were seeded on gold electrode-plated culture plates (8W10E+) and placed in an incubator at 37 °C with humidified air and 5% CO_2_. The attachment and spreading of the cells on electrodes was monitored continuously at 4 kHz until the observed electrical resistance reached a plateau and stabilized. This usually occurred within 20–24 h after inoculation. Impedance to current flow was measured at different frequencies (25 Hz to 60 kHz) periodically before and after inoculation. After reaching the steady-state of electric-cell substrate resistance, cells were exposed to the ROCK inhibitors, and the impedance was assessed at different frequencies every 30 min. The change in the measured resistance normalized to that of the bare electrode was taken as a measure of cell-matrix adhesion as discussed further below.

### Data analysis

A one-way ANOVA was used to compare mean values for different treatments with Bonferroni’s post-test analysis (Prism 5.0 for Windows; GraphPad Software, Inc., San Diego, CA). Results are expressed as mean±SEM. For analysis of ECIS (electrical cell-substrate impedance sensing) data, normalized numerical values from individual experiments were pooled and expressed as mean±SEM. A p value of less than 0.05 was considered statistically significant. “n” denotes the number of independent experiments performed. The IC_50_ values were determined by fitting the normalized response to the curve Y=100/(1+10^((X-LogIC_50_))) where Y denotes the normalized response and X is the log[concentration] of the drug. The calculations assume that log [drug] versus response follow a sigmoidal shape and were performed with Prism 5.0. The reported values of R indicate goodness of fit.

## Results

### Expression of myosin II, ROCK, MLCK, and MYPT isoforms

**Figure 1:**
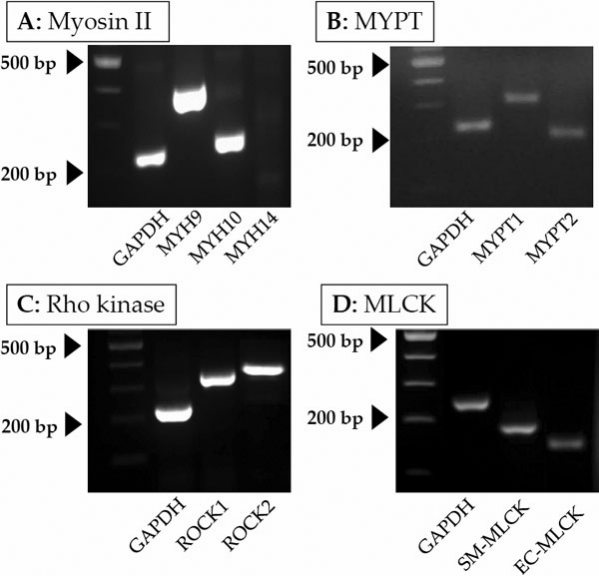
Expression of myosin II, *MYPT*, *ROCK*, and *MLCK* isoforms in GTM3 cells. Expression pattern of genes in cultured cells was determined using total RNA- and sequence-specific oligonucleotide primers. **A**: These cells express two isoforms of myosin II, namely, *MYH9* and *MYH10*. The other isoform, *MYH14*, was undetectable. In addition, the expression of both isoforms of *MYPT*, *ROCK*, and *MLCK* (**B**, **C**, **D**) was confirmed. Abbreviations: *MYH9*: myosin IIA; *MYH10*: myosin IIB; *MYH14*: myosin IIC; *ROCK*: Rho kinase; *MYPT*: myosin phosphatase; *MLCK*: myosin light chain kinase.

The GTM cell line was developed from a transformed strain of TM cells obtained from a glaucomatous donor [22]. Various pharmacological and morphological aspects of the cell line and comparison with normal human TM cells have been documented [22]. There were significant similarities between the two, as shown by the expression of specific cytoskeletal proteins, including tubulin, vimentin, α-SMA, and those associated with the ECM [22]. To facilitate our study on actomyosin contraction, we examined the expression of different isoforms of important actin cytoskeletal proteins at the mRNA level. As shown in Figure 1A, two isoforms of myosin II, namely, myosin IIA (*MYH9*) and myosin IIB (*MYH10*), are expressed in the GTM cells. We failed to detect the expression of myosin IIC (*MYH14*). The known isoforms of *MYPT* (*MYPT1* and *MYPT2*) and *ROCK* (*ROCK-I* and *ROCK-II*) were also expressed, along with the two *MLCK* splice variants (i.e., EC- and SM-MLCK; Figure 1B-D).

### Effect of ROCK inhibitors on MYPT1

**Figure 2:**
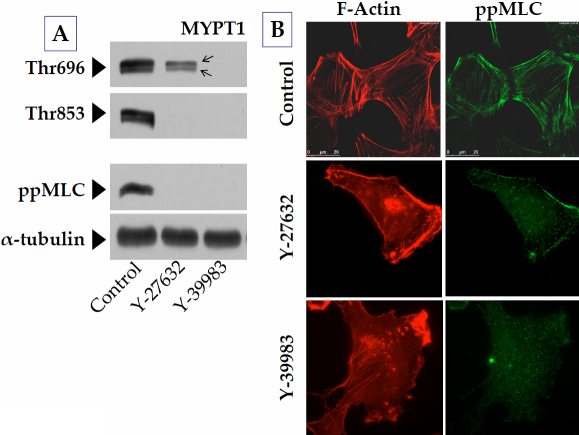
Effect of ROCK inhibitors on the phosphorylation of MYPT1 and MLC. Cells were treated with 5 μM of the specific ROCK inhibitors, Y-27632 and Y-39983, for 1 h in a serum-rich medium. **A**: Treatment with ROCK inhibitors completely opposed the phosphorylation of MYPT1 at Thr853. There was a concomitant decrease in the phosphorylation of MLC. However, the phosphorylation of MYPT1 at Thr696 was only slightly reduced in the presence of Y-27632 compared to Y-39983. **B**: In the presence of ROCK inhibitors, there was a loss in stress fibers, and the staining for MLC appeared diffuse.

Consistent with the mRNA expression, *MYPT1* expression in GTM cells was also noted at the protein level (Figure 2A). The two bands indicated by arrows in Figure 2A possibly correspond to the splice variants of 133 kDa and 130 kDa (shown by arrows in Figure 2A) [25]. Furthermore, as shown in Figure 2A, exposure to 5 μM of Y-27632 or Y-39983 led to the complete dephosphorylation of MYPT1 at Thr853. However, in contrast to Y-39983, Y-27632 induced a comparatively smaller reduction in MYPT1 phosphorylation at Thr696 (Figure 2A; middle lane). We next characterized the impact of the ROCK inhibitors on the dephosphorylation of MLC as a direct measure of the phosphatase activity of MLCP. In agreement with the impact on MYPT1 phosphorylation sites, both the ROCK inhibitors reduced MLC phosphorylation (diphospho form; denoted as ppMLC; Figure 2B). Immunocytochemistry data also showed that the intensity of ppMLC staining along F-actin was reduced. In addition, there was a loss of stress fibers in response to Y-27632 and Y-39983 (Figure 2B; middle and bottom rows).

**Figure 3:**
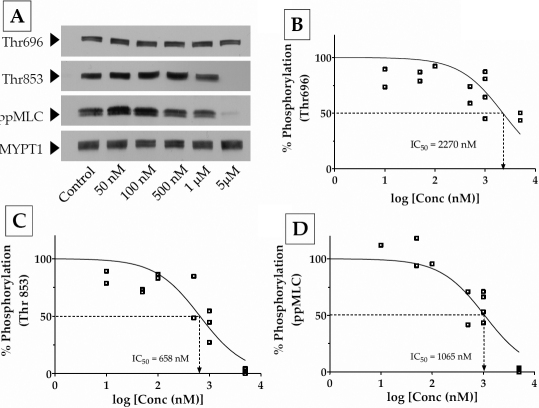
Concentration response to Y-27632. Confluent cells were treated with Y-27632 (10 nM to 5 μM) for 1 h in serum-rich medium. **A**: Representative data of the dose response of MYPT1 and MLC phosphorylation to Y-27632 (50 nM to 1 μM). There was only a marginal decrease in the phosphorylation of MYPT1 at Thr696, even at a drug concentration of 5 μM. **B**, **C**, and **D**: Densitometric analysis of western blot data shown in panel **A**. The IC_50_ of MYPT1 phosphorylation at Thr696 (IC_50_=2270 nM; **B**) is three times that of Thr853 (IC_50_=658 nM; **C**). The data obtained for phosphorylation at Thr696 were less repeatable and consistent (R^2^=0.39) compared with that obtained for Thr853 and MLC (R^2^=~0.8). However, there is a better correlation between MYPT1 dephosphorylation at Thr853 and MLC dephosphorylation (**D**) with near complete inhibition of both at a concentration of 5 μM.

**Figure 4:**
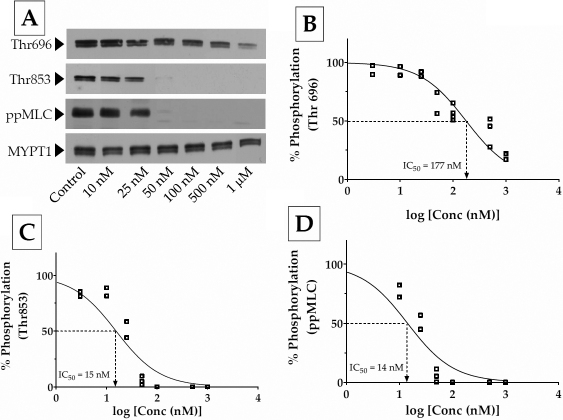
Concentration response to Y-39983. Confluent cells were treated with Y-39983 (3 nM to 5 μM) for 1 h in serum-rich medium. **A**: A typical dose response of MYPT1 and MLC phosphorylation to treatment with Y-39983 (50 nM to 1 μM). **B**, **C**, and **D**: Densitometric analysis of western blot data shown in panel **A**. **B**: There was a dose-dependent decrease in the phosphorylation of MYPT1 at Thr696 (IC_50_=177 nM). **C**: The inhibition of MYPT1 phosphorylation at Thr853 assumes a sigmoid curve compared with Thr696. There is a much steeper decrease in Thr853 phosphorylation with an IC_50_ of 15 nM. **D**: The dephosphorylation of MLC followed Thr853 closely with an IC_50_ of 14 nM.

To characterize further the differential sensitivity of MYPT1 phosphorylation sites to the ROCK inhibitors, we determined their IC_50_ values (i.e., concentration required to reduce phosphorylation of MYPT1 by 50%). Figure 3A is a typical concentration response of the dephosphorylation of MYPT1 and ppMLC to Y-27632 (10 nM to 5 μM). The IC_50_ for dephosphorylation at Thr696 for Y-27632 was 2270 nM (Figure 3B) in contrast to ~660 nM for dephosphorylation at Thr853 (Figure 3C). The latter was close to the IC_50_ for dephosphorylation of MLC (1065 nM; Figure 3D). Similar experimental results with Y-39983 are shown in Figure 4. Unlike Y-27632, Y-39983 showed an IC_50_ of ~200 nM for dephosphorylation at Thr696 (Figure 4B) and only 15 nM for dephosphorylation at Thr853 (Figure 4C). The IC_50_ for dephosphorylation of MLC was 14 nM (Figure 4D), which was close to the IC_50_ for Thr853 (Figure 4D). Taken together, these results show that Thr853 is the preferred site for the ROCK-mediated inhibition of MLCP. In addition, the data also indicates that Y-39983 is more potent than Y-27632 at inhibiting the influence of ROCK on MLCP activity.

### Effect of ROCK inhibitors on actomyosin contraction

**Figure 5:**
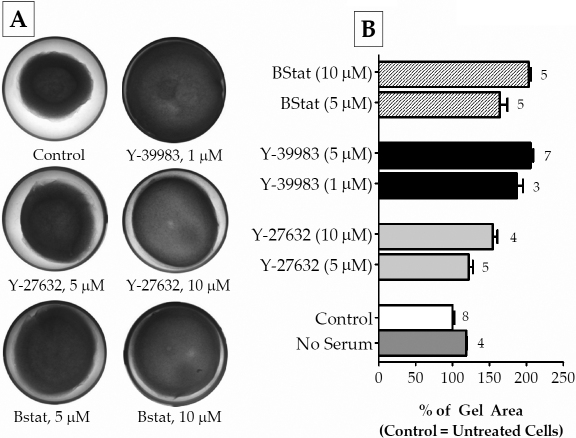
Effect of ROCK inhibitors on collagen gel contraction. Cells grown in collagen gels were incubated without (control) or with ROCK inhibitors Y-27632 (5 μM or 10 μM), and Y-39983 (1 μM or 5 μM) for 48 h. Changes in the area of the gels were calculated using the ImageJ program from NIH. The initial area of the gels (measured before the addition of drugs) was ~140 mm^2^. **A**: The presence of serum contracted the gels significantly. This serum-induced contraction was opposed by both Y-27632 and Y-39983, with the latter having a more significant effect. Blebbistatin (10 μM), a specific myosin II ATPase inhibitor, was employed to confirm that the observed changes in the gel area resulted from increased actomyosin contraction. **B**: A bar graph of the results from several independent experiments. As can be noted, the absence of serum led to a lesser contraction (by 18±0.5%) of the gels compared to serum (~60 mm^2^) after 48 h. The ROCK inhibitors significantly opposed the decrease in the gel area. At 10 μM Y-27632, the gel area had increased by 50%, while, even at 1 μM, Y-39983 opposed actomyosin contraction more effectively (86±5%) compared with the control. The gel area obtained with 10 μM of blebbistatin (denoted as Bstat) was similar to that obtained with 5 μM Y-39983. The data are represented as mean±SEM.

To evaluate further the effects of the ROCK inhibitors on MYPT1, we examined their potential downstream effects. As a measure of the direct effect of MYPT1, we performed collagen gel-contraction assays to assess actomyosin contraction, which is dependent on MLC phosphorylation. For this purpose, we first formed collagen gels showing an average area of 140 mm^2^ 1 h after polymerization. In the presence of 10% serum, GTM cells at a density of 2×10^5^ cells/ml led to a reproducible reduction in the area of the gels to ~60 mm^2^ in 48 h (Figure 5A,B). In the absence of serum, gels contracted 18±0.5% less compared with the presence of serum (n=4). When the cells were treated with Y-39983 for 48 h, there was an increase in the gel area by 86±5% (1 μM; n=3) and 105+1.2% (5 μM; n=7) compared with the control (Figure 5B). This significant relaxation in the presence of Y-39983 was much more than that obtained in response to Y-27632. Compared with the two ROCK inhibitors, a selective myosin II ATPase inhibitor, blebbistatin (10 μM), induced relaxation by 102±1.3% (n=5). Interestingly, this was similar to the efficacy of Y-39983 at 5 μM (Figure 5A,B).

### Effect of ROCK inhibitors on cell-matrix adhesion: Changes in electrical cell substrate resistance

We examined the cell-matrix adhesion as a second measure of the impact of ROCK inhibitors on MYPT1. It is well known that actomyosin contraction and cell-matrix adhesion possess a reciprocal relationship through their linkage via integrins [26]. We directly assessed cell-matrix adhesion using electric cell-substrate impedance sensing (ECIS; see Methods) and then indirectly characterized it in terms of the tyrosine phosphorylation of key proteins found at the focal adhesion sites.

**Figure 6:**
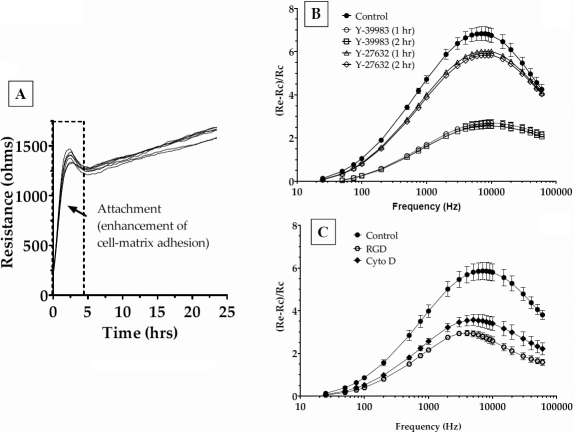
Cell-substrate impedance sensing. **A**: The evolution of resistance after seeding TM cells. Upon inoculation, the resistance measured at 4 kHz increased within 3 h. This was followed by a gradual increase in resistance (around 1600 ohms), which reached a plateau around 24 h. **B**: The impedance changes induced by ROCK inhibitors. To measure the changes in the measured resistance, frequency scans were taken every 30 min to obtain resistance values across 23 frequencies (23 Hz to 60 kHz) from each well. Treatment of cells with 5 μM Y-27632 or 1 μM Y-39983, denoted by closed squares and broken lines, respectively, led to a significant decrease in resistance. The decrease in resistance started at 30 min, reached a maximum at 1 h, and remained constant until 2 h after treatment. **C**: Treatment of cells with cytochalasin D (0.125 mg/ml; closed diamonds), an actin-depolymerizing agent, led to a significant decrease in the measured resistance. Similarly, treatment with an RGD-peptide (100 nM; a specific integrin binding peptide) led to a significant decrease in resistance (dashed lines) compared with the control (closed circles). Data shown are expressed as mean±SEM of three independent experiments.

ECIS involves the measurement of impedance to a small AC current applied across cells grown to confluence on planar gold microelectrodes [27,28]. The measurement is non-invasive, and off-the-shelf instruments such as ECIS^TM^ 1600R (Applied Biophysics, Inc., Troy, NY) make use of a lock-in amplifier to measure small changes in electrical impedance at a high-temporal resolution. We have previously employed ECIS to assess the integrity of the apical junctional complex of the corneal endothelial monolayers [29-31]. For epithelial monolayers, such as the corneal endothelium, the resistive component of the measured impedance implies trans-endothelial electrical resistance for current flow between the cells when confluent on the electrode [29-32]. However, with non-epithelial cells, such as fibroblasts, measured impedance arises mainly out of resistance to current flow across the cell substrate (i.e., cell-matrix adhesion) [33,34]. In this study, we examined the dynamics of such cell-matrix resistance (i.e., resistive component of the measured impedance) with TM cells. After seeding GTM cells on gold electrodes, the measured resistance with AC current at 4 kHz showed a steep peak in resistance, reaching ~1400 Ω within 3 h (Figure 6A). This increase can be attributed to the attachment of cells to the substrate (gold electrode), which leads to an increased resistance to current flow. Following this first phase, there is a gradual increase in the resistance to ~1600 Ω, reaching a steady-state at ~24 h (Figure 6A).

After the observed ECSR reached a steady-state, cells were challenged with the ROCK inhibitors, and the time-dependence of the ECSR was followed at several frequencies (Figure 6B). These frequency scans were intended to identify the frequency at which the altered ECSR would be most sensitive. It is apparent from these figures that the most sensitive frequency for TM cells is 7 kHz. Treatment with 5 μM of Y-27632 for 2 h led to a significant decrease in the peak resistance compared with untreated cells (Figure 6B). y-axis represents the change in ECSR normalized to the resistance of the corresponding bare electrode. Although the maximum decrease was attained after 1 h of treatment, the reduction in ECSR remained constant for up to 2 h. Treatment with Y-39983 at 1 μM led to a similar decrease in ECSR, showing a large decrease by 30 min at the same peak frequency and reaching a constant at 1 h. Unlike Y-27632, the response to Y-39983 was rapid and more pronounced (Figure 6B). Exposure to cytochalasin D, a known actin-depolymerizing agent, or an integrin-binding RGD peptide (cyclo-RGD peptide from Anaspec, Inc. [Fremont, CA], at 100 nM) also led to a substantial decrease in the resistance (Figure 6C).

### Effect of the ROCK inhibitors on cell-matrix adhesion: Changes in cell shape and focal adhesion

**Figure 7:**
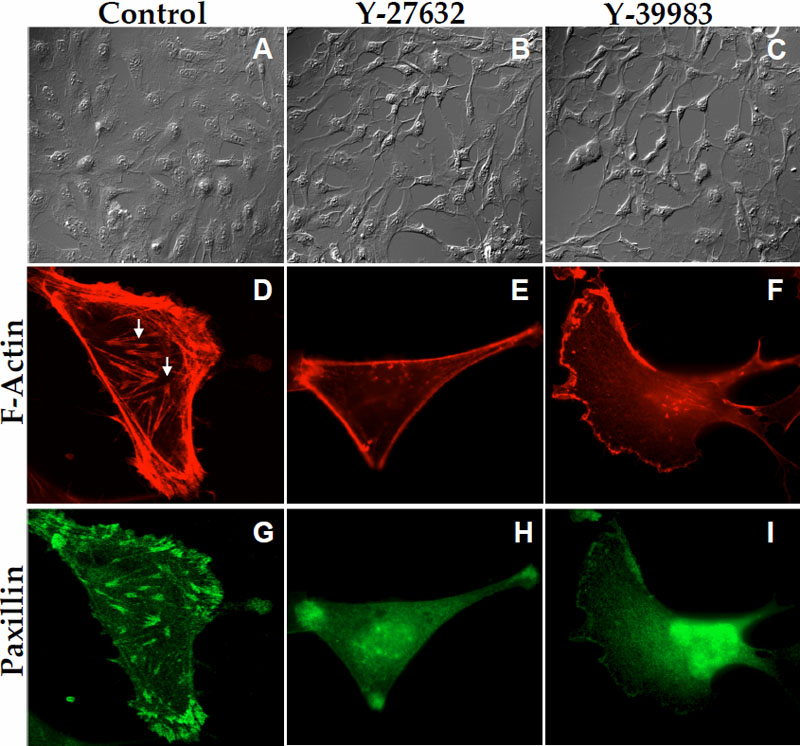
Effect of ROCK inhibition on cell morphology and focal adhesions. Cells grown on coverslips were treated with 5 μM Y-27632 and 1 μM Y-39983 for 1 h in serum-rich medium and stained for paxillin and F-actin. DIC images of these cells were also taken. **A**: The DIC image of untreated cells, which are spread out and have a flat appearance. **B** and **C**: Treatment with Y-27632 (5 μM) and Y-39983 (1 μM) for 1 h led to an apparent shrinkage and retraction of the cells, which assumed a stellate appearance. **D**: In untreated cells, prominent stress fibers are visible. Paxillin is seen as large spots within and at the cell boundaries (**G**). Treatment with Y-27632 (**E** and **H**) and Y-39983 (**F** and **I**) led to a complete loss of stress fibers and focal adhesions.

Actomyosin contraction affects cell shape and cell-matrix adhesion through its impact on the stress fibers. The altered cell-matrix adhesion manifests in part by tyrosine phosphorylation of focal adhesion proteins such as FAK, vinculin, and paxillin. Tyrosine phosphorylation is necessary for the functional assembly of the focal adhesion complexes and their association with the actin cytoskeleton [35]. The changes in cell shape in response to ROCK inhibitors are shown in the DIC images in Figure 7. When treated with Y-27632 (5 μM; Figure 7B) and Y-39983 (1 μM; Figure 7C) for 1 h, there was an apparent shrinkage, with cells assuming a stellate appearance compared to control.

**Figure 8:**
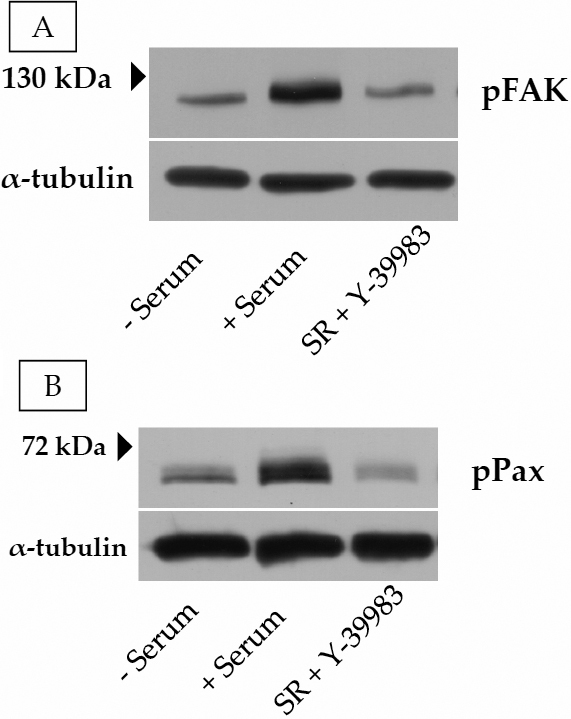
Influence of ROCK inhibition on phosphorylation of FAK and Paxillin. **A**: The addition of 10% serum to serum-starved cells led to a significant increase in the tyrosine phosphorylation of FAK tyrosine at residue Tyr397 (middle lane). This increase was opposed by pretreatment with Y-39983 (1 μM; last lane). **B**: Similarly, the serum-induced phosphorylation of paxillin at Tyr118 (middle lane) was also opposed by Y-39983 (last lane).

The disposition of the stress fibers with and without the ROCK inhibitors is shown in Figure 7, along with changes in tyrosine phosphorylation of the focal adhesion proteins (Figure 8). In untreated cells, as shown in Figure 7D, stress fibers are found along the cell periphery and across the cell body (shown by arrows). We have also shown the staining for focal adhesion protein paxillin in Figure 7G. Consistent with the strong adhesion in untreated cells, there was intense punctuate staining for the adhesion protein along the stress fibers. When cells were treated with the ROCK inhibitors, a loss in stress fibers at the periphery and across the cell body was apparent (Figure 7E,F). In addition, staining for paxillin became less prominent compared with control (Figure 7H,I). All the above responses were also observed when cells were treated with integrin-binding RGD peptide (data not shown).

Consistent with the immunofluorescence data, tyrosine phosphorylation of FAK and paxillin were altered in the presence of ROCK inhibitors (data for Y-27632 not shown). The addition of 10% serum to cells led to an increase in tyrosine phosphorylation of FAK (Tyr397; Figure 8A) and paxillin (Tyr118; Figure 8B). The ROCK inhibitors opposed this serum-induced increase in tyrosine phosphorylation of the adhesion proteins (Figure 8A,B). Taken together, the data in Figure 6, Figure 7, and Figure 8 indicate that loss in actomyosin contraction results in altered cell morphology due to weakening of primarily the cell-ECM adhesions.

## Discussion

**Figure 9:**
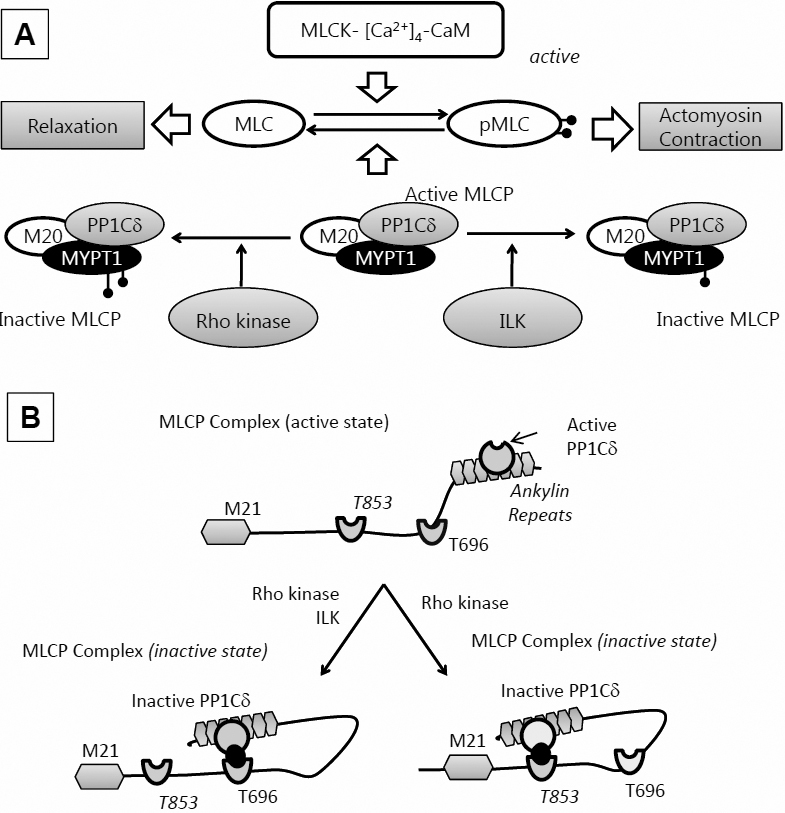
Cell signaling underlying the regulation of actomyosin contraction. **A**: MLC phosphorylation: MLC kinase (MLCK) drives phosphorylation and MLC phosphatase (MLCP) induces dephosphorylation. MLCP is a trimeric complex consisting of a phosphatase (PP1cδ), a myosin-binding subunit (MYPT1), and a subunit of unknown function (M20). MLCP activity is regulated by kinases like ROCK and integrin-linked kinase (ILK) through phosphorylation of MYPT1 at specific sites. This inactivates the catalytic subunit, thus preventing MLC dephosphorylation. **B**: Autoinhibition of MLCP: The substrate site of PP1cδ is accessible when neither Thr696 nor Thr853 is phosphorylated. However, when MYPT1 is phosphorylated at Thr696 or Thr853, the phosphorylated residues interact with the active site of PP1cδ and suppress the phosphatase activity. While both ROCK and ILK are known to phosphorylate Thr696, phosphorylation at Thr853 seems to be regulated exclusively by ROCK.

In humans, more than 80% of aqueous humor exits the anterior chamber via the TM route [36-38]. Therefore, outflow facility across TM is the primary determinant of IOP. Apart from pilocarpine, there are no drugs that elicit significant IOP reduction by enhancing outflow across TM without significant side effects. Prostaglandin analogs, the most efficacious ocular hypotensive drugs to date, mainly lower IOP by increasing outflow via the uveoscleral pathway [39]. Clearly, there is a challenge to find novel pharmaceutical agents to modulate the TM to elicit further reductions in IOP. Recent studies have gathered significant evidence to suggest that it is possible to manipulate the actin cytoskeleton of TM cells to alter the aqueous outflow facility. As noted earlier, drugs that enhance actomyosin contraction are known to increase resistance to outflow facility and vice versa [5,40,41]. Although a mechanistic link between actin cytoskeleton and outflow facility is not well understood, a dynamic ECM involvement seems likely. To delineate this phenomenon, it is imperative to establish the signaling underlying actomyosin contraction in TM cells and its coupling to the ECM. In this study, we set out to distinguish the two specific phosphorylation sites (Thr853 and Thr696) on MYPT1 that are thought to be central to actomyosin contraction via the RhoA-Rho kinase axis. Specifically, we investigated the differential action of two ROCK inhibitors on the phosphorylation of MYPT1 at the two sites. Both the inhibitors have been reported to increase outflow facility across the TM in ocular perfusion studies and in vivo in animal models and to lower IOP [19-21,42]. Our major findings indicate that the effect of ROCK inhibitors is predominantly at Thr853 of MYPT1 and that consequent loss of actomyosin contraction weakens the cell-matrix adhesion and alters cellular morphology. Thus, our results suggest a distinct molecular target to assess the impact of potential ROCK inhibitors or other mechanisms of RhoA-Rho kinase axis antagonism on TM cells (summarized in Figure 9).

### Characterization of GTM3 cells

In the context of our specific objectives outlined above, we performed our experiments with a TM cell line (i.e., GTM3 cells) developed previously from a human donor with glaucoma [22]. As noted earlier, these GTM3 cells are similar to normal human TM cells with a similar expression profile of key structural proteins and characteristic cell signaling responses to several G-protein coupled receptor (GPCR) agonists [22]. As an extension of the earlier experiments, we characterized GTM3 cells by determining the expression profile of proteins involved in the regulation of actomyosin contraction. As in smooth muscle cell types, GTM cells were found to express different isoforms of the myosin II, *MLCK*, *MYPT*, and *ROCK* genes. The expression of the short isoform of *MLCK* was first reported in smooth muscle cells (hence, called *SM-MLCK*), while the longer splice variant (*EC-MLCK*) was found in vascular endothelial cells and other non-smooth muscle cell types (e.g., corneal endothelium) [43]. This expression pattern is similar to that of bovine TM cells [44]. The expression of *ROCK* isoforms (*ROCK-I* and *ROCK-II* isoforms) is not unique to GTM cells, as their expression has been reported in the TM cells derived from bovine, monkey, and human sources [20,42,45]. A recent study by Nakajima et al. [42] showed the expression of *MYPT1* and *MYPT2* isoforms in monkey and human TM, and hence the expression in GTM cells, was also as expected. Expression of myosin II isoforms *MYH9* and *MYH10*, but not of *MYH14*, has been previously reported in normal TM cells [46]. The expression pattern of these elements in GTM3 cells was the same as in normal TM cells. Taken together, the gene expression pattern of the key proteins involved in actomyosin contraction was similar to that found in normal TM cells.

### MYPT1 phosphorylation sites and their differential sensitivity to ROCK inhibitors

Members of the MYPT family include separate gene products such as *MYPT1*, *MYPT2* (in striated muscle and brain), *MBS85*, *MYPT3*, and *TIMAP* [12]. *MYPT1* is expressed in multiple isoforms generated by alternative splicing showing differential sensitivity to cGMP/PKG [47]. The doublet of *MYPT1*, as seen in Figure 2A, is suggestive of the splice variants of 130 kDa and 133 kDa [25]. The shorter splice variant in chicken gizzard was shown to lack a central 123-nucleotide exon and therefore to be irresponsive to Ca^2+^ sensitization [48]. Several sites on MYPT1, susceptible to Ser/Thr phosphorylation, regulate the activity of PP1cδ, leading to the inhibition of MLCP [13]. As noted earlier, Thr696 and Thr853 of MYPT1 are known sites phosphorylated by ROCK, which elicit enhanced MLC phosphorylation through the inhibition of MLCP [14] (also see Figure 9). In a recent study, it was shown that the substrate site of PP1cδ, bound to the ankyrin repeat of MYPT1, is accessible when Thr696 and/or Thr853 are not phosphorylated. When phosphorylated, the residues interact with the active site of PP1cδ and suppress its phosphatase activity, leading to increased MLC phosphorylation [49].

Employing site-specific phospho antibodies, we examined the relative phosphorylation at Thr696 and Thr853 without and with ROCK inhibitors. As expected, both Y-27632 and Y-39983 reduced the phosphorylation at Thr696 and Thr853 and led to decreased MLC phosphorylation as well as reduced actomyosin contraction. It is evident from the IC_50_ values summarized in Table 2 that inhibition of phosphorylation at Thr853 by both the inhibitors is pronounced compared with that at Thr696. Moreover, the IC_50_ values for dephosphorylation of MLC were close to the IC_50_ values for dephosphorylation of MYPT1 at Thr853. This implies that PP1cδ activity, in other words, activity of MLCP, is most sensitive to the phosphorylation status of Thr853. In addition to differential sensitivity toward the site of phosphorylation, our data also demonstrates that Y-39983 is more potent compared with Y-27632. This is apparent in the IC_50_ values obtained for dephosphorylation at Thr853, Thr696, and pMLC and is consistent with a previous report that showed that Y-39983 is 30-fold more potent (IC_50_ of Y-39983 and Y-27632 for inhibiting ROCK was 3.6 nM and 110 nM, respectively) and specific at inhibiting ROCK compared to Y-27632 [19]. Further, at 5 μM concentration, Y-39983 led to a 100% decrease in the contraction of the collagen gels while at 10 μM concentration, Y-27632 led to only a 50% relaxation of the collagen gels (Figure 5). This observation is consistent with the results obtained with normal HTM cells wherein 10 μM of Y-27632 allowed only 30% gel contraction [23]. Finally, Y-39983 has been shown to lead to a 10-fold decrease in IOP compared to Y-27632 in rabbits [19].

**Table 2:** IC_50_ for inhibition of phosphorylation at Thr853 and Thr696 of MYPT1 by the Rho kinase inhibitors.

**Target/Function**	**Y-27632 (nM)**	**Y-39983 (nM)**
Thr853	658	15
Thr696	2270	177
MLC	1065	14
Relaxation	++	+++++

In addition to MYPT1 phosphorylation and inactivation, ROCK has been shown to directly phosphorylate recombinant MLC at Ser19 similar to MLCK [50]. However, this effect has not been demonstrated in vivo. On the contrary, it has been shown that the inactivation of the phosphatase activity is more important for Ca^2+^ sensitization [51]. Our data also agree with the later observation in that the IC_50_ values of MLC phosphorylation closely follow the IC_50_ values obtained for phosphorylation at Thr853. If there were direct phosphorylation of MLC by ROCK, we would not expect to see this agreement in the IC_50_ values.

### Effect of ROCK inhibitors on cell-matrix adhesion and morphology

A recent study on TM demonstrated that expression of constitutively active RhoA led to an increase in stress fibers, actomyosin contraction, focal adhesions, and the gene expression of various ECM proteins [52]. In the current study, relaxation caused by the inhibition of ROCK is characterized by retraction of the cells as a result of decreased cell-ECM interactions.

In the past, cell-ECM interactions have been studied using qualitative immunocytochemical techniques. Here, we have employed a sensitive technique, ECIS, to measure cell-ECM interactions. This technique has been previously used to study cell signaling mechanisms by monitoring the changes in cellular characteristics such as migration [53], cell-cell adhesions [30,32], and cell-ECM adhesions [33,54], and morphology [44,55,56]. As observed in this study, the initial attachment and spreading characteristics of the TM cells suggest that the majority of resistance to current flow is provided by the cell-ECM interactions as opposed to the cell-cell interactions. This observation is contrary to the findings in many epithelial and endothelial cells with typical tight junctions where the major resistance to current flow lies at the level of cell-cell junctions [57]. As shown in Figure 6, the inhibition of ROCK led to a significant decrease in the cell-substrate resistance indicating a change in cell-matrix adhesion. Similarly, the resistance obtained with an integrin-binding peptide and an actin-depolymerizing agent was decreased compared with untreated cells. These findings are corroborated by the immunofluorescence data, which show that treatment with the ROCK inhibitors and the peptide result in a loss in stress fibers and in the staining for the focal adhesion proteins paxillin. Finally, serum-induced tyrosine phosphorylation of the focal adhesion proteins, FAK and paxillin, was reduced to control levels by the ROCK inhibitors. These results confirm the reciprocal relationship between the actin cytoskeleton and focal adhesions and support the results reported by a recent publication wherein treatment with ROCK inhibitors was shown to reduce the impedance values of GTM cells [58]. However, the extent to which the change in electric resistance shown in Figure 6 is convolved with changes in morphology (Figure 7) is yet to be resolved.

The resistance to outflow facility through the TM is due to increased actomyosin contraction and/or alterations in the ECM. These factors are not mutually exclusive, but modulate each other. As discussed above, increased actomyosin contraction reduces the outflow facility. This is thought to lead to the compaction of the TM tissue, which leads to reduced intertrabecular pores and therefore an increase in the resistance to the flow of fluid. On the other hand, the increase in the deposition of ECM in the anterior chamber can in itself reduce TM porosity and obstruct the fluid flow. In addition, the change in the shape of the cells (retraction of cells as seen with agents that increase cAMP and disrupt actin polymerization and ROCK inhibitors) might increase the porosity of the TM and thus aid the egress of aqueous humor from the anterior chamber, leading to a reduction in IOP. Therefore, it is hoped that, along with the existing pharmaceutical strategies to treat ocular hypertension, suitable ROCK inhibitors with adequate efficacy may soon become available to treat ocular hypertension and glaucoma. In a recent study [44], we have demonstrated that elevated intracellular cAMP opposes MLC phosphorylation and leads to loss of actomyosin contraction by reducing the phosphorylation of MYPT1 at Thr853 similar to Rho kinase inhibitors. We also observed by electric cell substrate impedance measurements that by relaxing actomyosin contractility, cAMP opposes cell-matrix adhesion in manner similar to Rho kinase inhibitors.

In light of suitable drug development, this study highlights an important signaling mechanism through which ROCK inhibits the phosphatase activity, leading to increased actomyosin contraction. Specifically, the results highlight the difference in the sensitivities of the two phosphorylation sites on MYPT1 to ROCK inhibition with the phosphorylation at Thr853 regulating the phosphorylation of MLC (summarized in Figure 9). Our ability to establish that Y-39983 is more potent than Y-27632 at inhibiting phosphatase activity based on the phosphorylation status of Thr853 suggests that this can be used as a reliable substrate for establishing the potency of ROCK inhibitors. With the availability of high throughput western blot analysis systems [59], and cellular impedance measurements [58], this knowledge can be used in the screening for potential ROCK inhibitors.
